# Hypoxia Activates the PTHrP –MEF2C Pathway to Attenuate Hypertrophy in Mesenchymal Stem Cell Derived Cartilage

**DOI:** 10.1038/s41598-019-49499-x

**Published:** 2019-09-16

**Authors:** David C. Browe, Cynthia M. Coleman, Frank P. Barry, Stephen J. Elliman

**Affiliations:** 1 Regenerative Medicine Institute, National University of Ireland Galway, University Road, Galway, Ireland; 2 Orbsen Therapeutics Ltd, National University of Ireland Galway, Distillery Road, Galway, Ireland

**Keywords:** Mesenchymal stem cells, Mesenchymal stem cells

## Abstract

Articular cartilage lacks an intrinsic repair capacity and due to the ability of mesenchymal stem cells (MSCs) to differentiate into chondrocytes, MSCs have been touted as a cellular source to regenerate damaged cartilage. However, a number of prevailing concerns for such a treatment remain. Generally, administration of MSCs into a cartilage defect results in poor regeneration of the damaged cartilage with the repaired cartilage consisting primarily of fibro-cartilage rather than hyaline cartilage. Methods that improve the chondrogenic potential of transplanted MSCs *in vivo* may be advantageous. In addition, the proclivity of MSC-derived cartilage to undergo hypertrophic differentiation or form bone *in vivo* also remains a clinical concern. If MSC-derived cartilage was to undergo hypertrophic differentiation *in vivo*, this would be deleterious in a clinical setting. This study focuses on establishing a mechanism of action by which hypoxia or low oxygen tension can be used to both enhance chondrogenesis and attenuate hypertrophic differentiation of both MSC and ATDC5 derived chondrocytes. Having elucidated a novel mechanism of action, the subsequent goals of this study were to develop an *in vitro* culture regime to mimic the beneficial effects of physiological low oxygen tension in a normoxic environment.

## Introduction

Mesenchymal stem cells (MSC) derived from human bone marrow (BM) and adipose tissue are currently being evaluated in early phase clinical trials to treat osteoarthritis (OA) and cartilage defects^1,2^. MSCs are an ideal cell source to treat cartilage disorders as MSCs are easily obtained, exhibit a high expansion potential, and retain the potential to differentiate along chondrogenic lineages^3^. However, MSC-derived cartilage has the proclivity to undergo hypertrophic differentiation. This hypertrophy resembles endochondral ossification: a cartilage precursor before matrix mineralization and ossification^4^. Previous studies have revealed that MSC micromass chondrogenic pellets undergo hypertrophy and were mineralised upon subcutaneous transplantation into severe combined immunodeficiency (SCID) mice^5,6^. This tendency of MSC derived cartilage to undergo hypertrophy and form bone is also being explored for use in tissue engineering approaches for bone repair^7–11^. Early clinical safety studies using autologous MSCs to treat cartilage defects resulted in an improvement in patient pain and joint function. However, long-term follow up examination of the *de novo* tissue revealed a “hyaline-like” cartilage which was deemed to be biomechanically inferior to healthy cartilage^12,13^. Therefore, the development of a therapeutic MSC product that both resembles native articular cartilage and is resistant to hypertrophy remains a challenge for in the field of tissue engineering.

In order to examine the molecular mechanism regulating attenuation of hypertrophy, this study will employ the use of the chondrogenic cell line ATDC5 in addition to primary human bone marrow derived- MSCs. The ATDC5 cell line was established by Atsumi and colleagues in 1990^14^. ATDC5 cells were derived from and isolated from murine teratocarcinoma fibroblastic cells and were observed to undergo high levels of chondrogenic differentiation in comparison to other cells lines that are also used to study 2D chondrogenesis such as C3HT10½ cells^14^. ATDC5 cells have been shown to demonstrate sequential chondrogenic differentiation, in such that the cells will deposit GAG and ECM followed by the upregulation of hypertrophy markers such as collagen X^15–17^. ATDC5 cells are both a stable cell line and are not detrimentally effected by passage as is the case with primary cells, these properties make ATDC5 cells an ideal cell source to study the molecular mechanisms of chondrogenesis^18^.

Hypoxia, specifically the induction of hypoxia inducible factor 2α (HIF2α), can support chondrogenesis via increased expression of the transcription factor, SRY (sex determining region Y) -box 9 (SOX9) and Collagen-type 2 (Col2a1)^19–22^. Additionally, hypoxia causes decreased expression of hypertrophic markers Collagen X and Runt-related Transcription Factor 2 (RUNX2) during chondrogenesis, however the mechanism of action has yet to be fully elucidated^23–25^. We were interested in identifying factors involved in the hypertrophic differentiation of cartilage with the aim of discovering pharmacological inhibitors of this process. It has been previously reported that human articular chondrocytes cultured in hypoxia exhibit enhanced Parathyroid hormone related protein (PTHrP) expression in a HIF1α- and HIF2α-dependent manner^26^. PTHrP is a secreted protein that maintains cartilage homeostasis and plays a pivotal role during skeletal development by inhibiting hypertrophic differentiation of chondrocytes^27^. Additionally, treatment of MSC-derived cartilage with recombinant PTHrP peptide resulted in a reduction of hypertrophy markers/mediators Collagen X and Alkaline phosphatase (ALP)^6,28^. However, PTHrP peptides also caused a decrease in Collagen II deposition, indicating a reduction in chondrogenesis which would be deleterious in a therapeutic setting^6,28^. The anti-hypertrophic effects of PTHrP are mediated by the receptor, PTHR1^29^. Upon activation of PTHR1 by PTHrP, a decrease in the transcriptional factor Myocyte enhancement factor 2C (MEF2C) supresses hypertrophy by reducing Col10a1 gene expression^30,31^. MEF2C is also involved in matrix mineralization by osteoblasts, where knockdown of MEF2C attenuates osteogenic/hypertrophic genes including RUNX2 and matrix metalloproteinase 13 (MMP13)^32^.

We hypothesize that activation of hypoxia inducible pathways by physiological, genetic or pharmacological means can have beneficial effects on the development of cartilage formed. It does so by attenuating hypertrophy and thus improving the phenotype of the *de novo* cartilage so it resembles native articular cartilage. We aim to demonstrate that this effect is as a result of a hypoxia-induced stimulation of a PTHrP – MEF2C pathway and subsequent repression of hypertrophic markers/mediators. Secondly, we aim to demonstrate the development of an *in vitro* culture regime to mimic the beneficial effects of physiological low oxygen tension in a normoxic environment. We propose that such a regime could be clinically translated to *in vivo* settings to promote the formation of *de novo* cartilage that closely resembles native articular cartilage.

## Results

### Hypoxia enhances chondrogenesis in human MSC micromass pellets and murine ATDC5 monolayer differentiation

**Figure 1:**
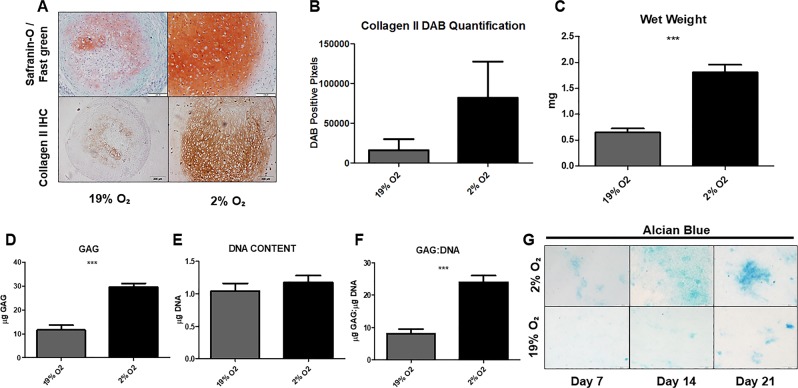
Hypoxia improves chondrogenesis in MSC micromass pellets and ATDC5 cultures. Human MSC micromass pellets were differentiated in hypoxia or normoxia for 28 days (**A–F)**. An increase in positive staining for GAG in pellets differentiated in hypoxia was observed following Safranin-O histological staining (**A**). Immunohistochemistry for Collagen II deposition revealed an upregulation in Collagen II protein deposition in pellets differentiated in hypoxia when compared to normoxia (**A**). Quantification of DAB positive pixels demonstrates a 5-fold increase in Collagen II under hypoxic conditions when compared to normoxia (**B**). Hypoxia significantly increased the wet weight of MSC micromass pellets (p = < 0.001) (**C**). DMMB and Pico Green assays were performed on papain-digested MSC micromass pellets: hypoxia significantly increased both the GAG (p = < 0.001) (**D**) and GAG:DNA ratio (**F**). Hypoxia had no effect on the DNA content of the MSC micromass pellets (**E**). ATDC5 were differentiated in mono-layer conditions for 7, 14 and 21 days in either hypoxia or normoxia. Alcian blue staining for GAG demonstrates an increase in GAG production as a result of hypoxia, positive staining was also observed at earlier time-points in hypoxic cultures (**G**). Scale bars = 200 µm.

To assess the effect of hypoxia upon MSC chondrogenesis, pellets were differentiated for 28 days in normoxic (19% O_2_) or hypoxic  (2% O_2_) conditions. An increased level of GAG in the hypoxia group was observed by Safranin-O staining of MSC pellets at 28 days. Pellets cultured in hypoxia displayed a more uniform distribution of GAG throughout the matrix, whereas in normoxic pellets positive GAG staining was isolated in the centre of the pellet (Fig. 1A).

Collagen II protein was increased in MSC pellets differentiated in hypoxic conditions when compared to the normoxic group, as shown by immunohistochemistry of histological sections from day 28 pellets (Fig. 1A). The location of the positive Collagen II staining correlates with positive GAG staining, with hypoxic pellets displaying strong, uniform staining throughout the pellets. By contrast, normoxic pellets displayed weak staining, which was confined to the centre of the pellet (Fig. 1A). Collagen II staining was quantified by measuring the number of DAB positive pixels in images from 3 donors; a 5-fold increase in Collagen II staining was observed in pellets cultured under hypoxic conditions (Fig. 1B).

Hypoxic differentiation also resulted in an increase in the mass of the pellets. MSC pellets cultured in hypoxia yielded a 3-fold increase in wet weight compared to normoxic controls (Fig. 1C).

GAG deposition was analysed by DMMB assay to quantify levels of sulphated GAG. By 28 days, pellets differentiated in hypoxia had deposited 2.5-fold more GAG than normoxic pellets (Fig. 1D). As no increase in the overall DNA content was found between the two treatments (Fig. 1E), a significant increase was demonstrated in the GAG:DNA ratio of the pellets differentiated in hypoxia, when compared to normoxic pellets (Fig. 1F).

In addition, to investigate if the response to hypoxia was restricted to human cells and/or a 3D culture regimen, we examined the effect of hypoxia on the monolayer differentiation of the murine cell line ATDC5. Under hypoxic conditions, ATDC5 differentiation results in an increase in Alcian blue positive cartilage nodules, when compared to the normoxia group, thus indicating increased GAG in the extracellular matrix (ECM). We observed that, at early time-points, hypoxia induces GAG deposition sooner during differentiation of ATDC5 than when compared to normoxic controls (Fig. 1G).

### Hypoxia attenuates hypertrophy

**Figure 2:**
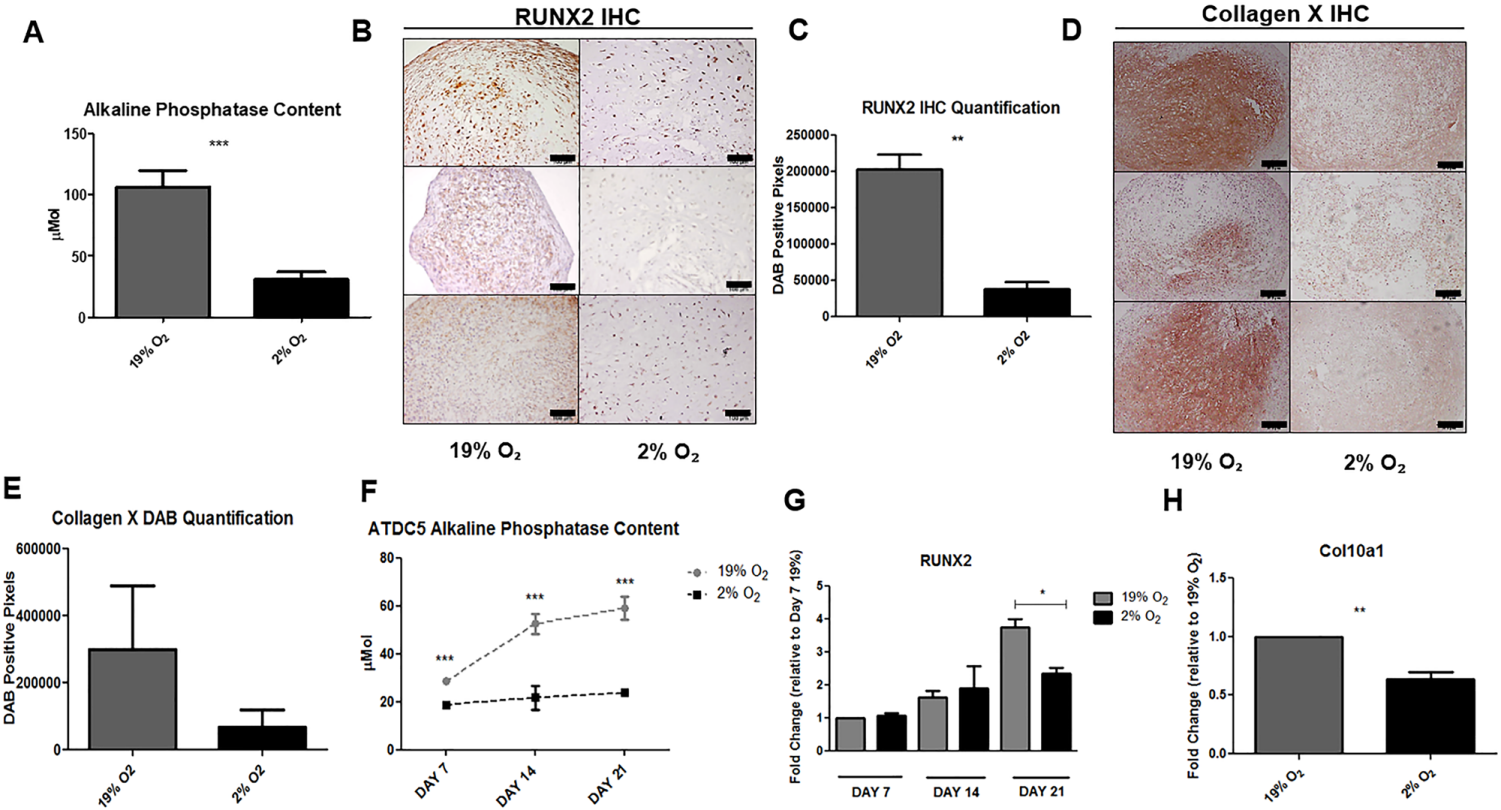
Hypoxia attenuates markers of hypertrophy. A pNPP assay was used to quantify ALP content in CM from day 28 MSC micromass pellets. A significant decrease in ALP content was observed in MSC micromass pellets differentiated in hypoxia when compared to normoxia controls (p = < 0.001) (**A**). Immunohistochemistry was performed on sections from day 28 MSC micromass pellets. A reduction in positive staining for RUNX2 (scale bar = 100 µm) (**B**) and Collagen X protein deposition (scale bar = 200 µm) (**D**) was observed in MSC micromass pellets differentiated in hypoxic conditions. Quantification of this reduction in DAB staining was confirmed following analysis of DAB positive pixels in all images (**C,E**). ALP content from CM of ATDC5 differentiated under hypoxic and normoxic conditions across a 21 day time course demonstrated significant decreases in ALP content in ATDC5 differentiated in hypoxia at all time points, when compared to normoxia (p = < 0.001) (**F**). QPCR for RUNX2 was performed on ATDC5 differentiated in hypoxia and normoxia for 21 days; at day 21 of differentiation ATDC5 differentiated in hypoxia had significantly reduced RUNX2 mRNA expression when compared to normoxic controls (p = 0.0447) (**G**). Col10a1 gene expression in ATDC5 was analysed at day 21 of differentiation by QPCR; hypoxia was found to significantly reduce Col10a1 gene expression when normalized to normoxic controls (p = 0.0036) (**H**). Scale bars = 200 µm.

We examined the effects of hypoxia on hypertrophy, as indicated by the decreased expression of RUNX2, Collagen X and ALP content. ALP content was reduced by 71% in the conditioned media (CM) of day 28 MSC pellets in hypoxia when compared to CM from MSC pellets differentiated in normoxia (Fig. 2A).

In human MSC pellets, hypoxia reduced expression of RUNX2 protein at day 28 when compared to pellets cultured in normoxia, as demonstrated by immunohistochemistry (Fig. 2B). Upon quantification, RUNX2 protein deposition was found to be significantly reduced by hypoxic differentiation (Fig. 2C). Additionally, a decrease in Collagen X protein deposition in day 28 MSC pellets was observed by immunohistochemistry when compared to normoxic controls (Fig. 2D). The reduction in Collagen X was further confirmed by quantification of Collagen X staining (Fig. 2E).

To examine if hypoxia attenuated hypertrophy in ATDC5 cells undergoing chondrogenesis, ATDC5 cells were differentiated in a monolayer for 21 days. ATDC5 cells differentiated in normoxia show elevated levels of ALP content when compared to ATDC5 cells differentiated in hypoxia, which maintain constant low levels of ALP content throughout differentiation (Fig. 2F).

To establish if hypoxia inhibited key regulators of hypertrophy, the expression of RUNX2 and Col10a1 was assessed. RUNX2 mRNA levels were significantly reduced by ~40% in ATDC5 differentiated for 21 days in hypoxia. No significant change in RUNX2 mRNA expression was observed on days 7 and 14 of differentiation (Fig. 2G). ATDC5 differentiated under hypoxic conditions exhibited significantly reduced gene expression of Col10a1 at day 21 of differentiation (Fig. 2H); under these conditions we were unable to detect Col10a1 gene expression at days 7 and 14 of ATDC5 differentiation.

### Hypoxia upregulates PTHrP expression during chondrogenesis

**Figure 3:**
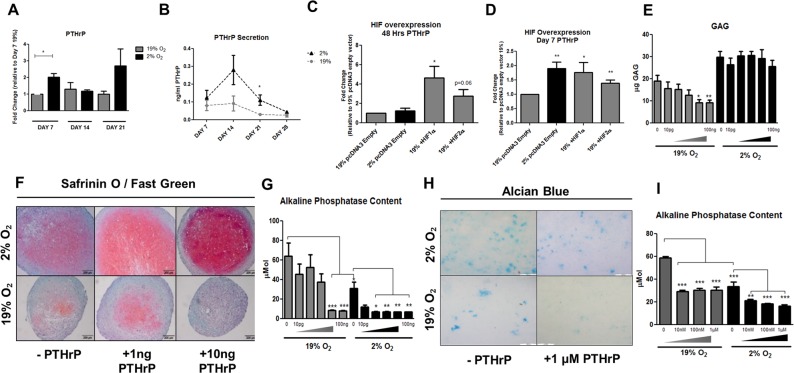
Hypoxia stimulates expression of PTHrP. Differentiation of ATDC5 under hypoxic conditions resulted in an upregulation of PTHrP mRNA at day 7 (p = 0.0107) and day 21 (p = 0.1756) of differentiation when compared to normoxic controls (**A**). Secretion of PTHrP into CM from MSC micromass pellets was quantified by ELISA across a 28 day time course. PTHrP protein secretion was elevated in hypoxia at day 14 (p = 0.09) and day 21 (p = 0.0399) of differentiation (**B**). Overexpression of HIF1α and HIF2α in ATDC5 resulted in an upregulation of PTHrP mRNA. 48 hours post treatment, PTHrP mRNA levels were significantly elevated in ATDC5 overexpressing HIF1α (p = 0.0358). HIF2α overexpression also increased PTHrP transcript levels but this effect was not significant (p = 0.0617) when compared to normoxic empty vector controls (**C**). 7 days post HIF overexpression, PTHrP mRNA levels were significantly increased in response to hypoxia alone (p = 0.0043), HIF1α overexpression (p = 0.0229) and HIF2α overexpression (p = 0.0032), when compared to normoxic empty vector controls (**D**). In a gain of function experiment, PTHrP (1–34) peptide was added three times per week to the differentiation media of MSC micromass pellets from day 14 to day 28 of differentiation. Under normoxic conditions, the addition of PTHrP peptide had a dose-dependent detrimental effect on GAG deposition as demonstrated by DMMB assay (**E**) and Safranin O / Fast Green staining (**F**). Increasing concentrations of PTHrP peptide had no negative effect on GAG deposition in MSC micromass pellets differentiated in hypoxia (**E**,**F**). ALP content was dose-dependently reduced by the addition of PTHrP peptide in MSC micromass pellets differentiated in normoxia, with 10 ng/ml (p = 0.0007) and 100 ng/ml (p = 0.0006) significantly reducing ALP content (**G**). In hypoxia, ALP content was significantly reduced when compared to normoxia controls at all concentrations, with a concentration of 100 pg/ml PTHrP peptide having a significant reduction when compared to non-treated hypoxic controls (**G**). In ATDC5, PTHrP peptide addition demonstrated similar effects to those seen in MSC micromass pellets with significant decreases in ALP content in response to PTHrP peptide addition; however, as with the MSC micromass pellets experiments, high concentrations of PTHrP peptide had a detrimental effect on ECM synthesis as observed by Alcian blue staining of ATDC5 (**H**,**I**). Scale bars = 200 µm.

Monolayer differentiation of ATDC5 cells under low oxygen conditions resulted in a 2 and 2.5 fold increase in PTHrP mRNA levels at days 7 and 21 respectively, when compared to levels under normoxic conditions. Comparable levels of PTHrP mRNA were observed in both hypoxic and normoxic groups at day 14 of differentiation (Fig. 3A). To quantify exogenous PTHrP (1–34) protein in response to hypoxia, CM was collected from MSC pellets differentiated in hypoxia or normoxia, over a 28 day time course. Increased levels of secreted PTHrP were detected at days 14 and 21 of hypoxic differentiation by ELISA (Fig. 3B).

HIF1α and HIF2α were overexpressed in ATDC5 differentiated under normoxic conditions to determine if hypoxic regulators could affect PTHrP levels during differentiation. At 48 hours post transfection, only HIF1α overexpression resulted in a significant upregulation in PTHrP mRNA levels when compared to empty vector controls (Fig. 3C). At 7 days post transfection, significant upregulation in PTHrP mRNA was observed in ATDC5 overexpressing HIF1α and HIF2α as well as empty vector hypoxic controls (Fig. 3D).

Having established that PTHrP is regulated by HIF and PTHrP is upregulated during hypoxic chondrogenesis we sought to examine the effects of exogenous PTHrP supplementation during chondrogenic differentiation. PTHrP (1–34) peptide was added to CCM in a range from 10 pg/ml to 100 ng/ml. During differentiation under normoxic conditions we observed a dose-dependent decrease in GAG production in MSC pellets with increased concentrations of PTHrP (1–34) peptide (Fig. 3E (GAG assay) and Fig. 3F (Safranin-O-staining)). Conversely, MSC pellets differentiated under hypoxic conditions showed no reduction in GAG synthesis as a result of PTHrP (1–34) peptide addition (Fig. 3E). No significant changes in DNA content were observed as a result of PTHrP (1–34) peptide treatment (data not shown). PTHrP (1–34) peptide also had a similar effect on the differentiation of ATDC5 cells: under normoxic conditions, 1 μM of PTHrP (1–34) had a detrimental effect on GAG, as indicated by reduced Alcian blue staining, which was not observed under hypoxic conditions (Fig. 3H). The addition of PTHrP (1–34) peptide during hypoxic and normoxic differentiation also caused a dose-dependent reduction in ALP content in MSC pellets (Fig. 3G) and ATDC5 cells (Fig. 3I). These data suggest the PTHrP (1–34) peptide attenuates chondrogenic hypertrophy under hypoxic and normoxic conditions in two cellular systems.

### Hypoxia attenuates hypertrophy via a PTHrP mediated reduction in MEF2C expression

**Figure 4:**
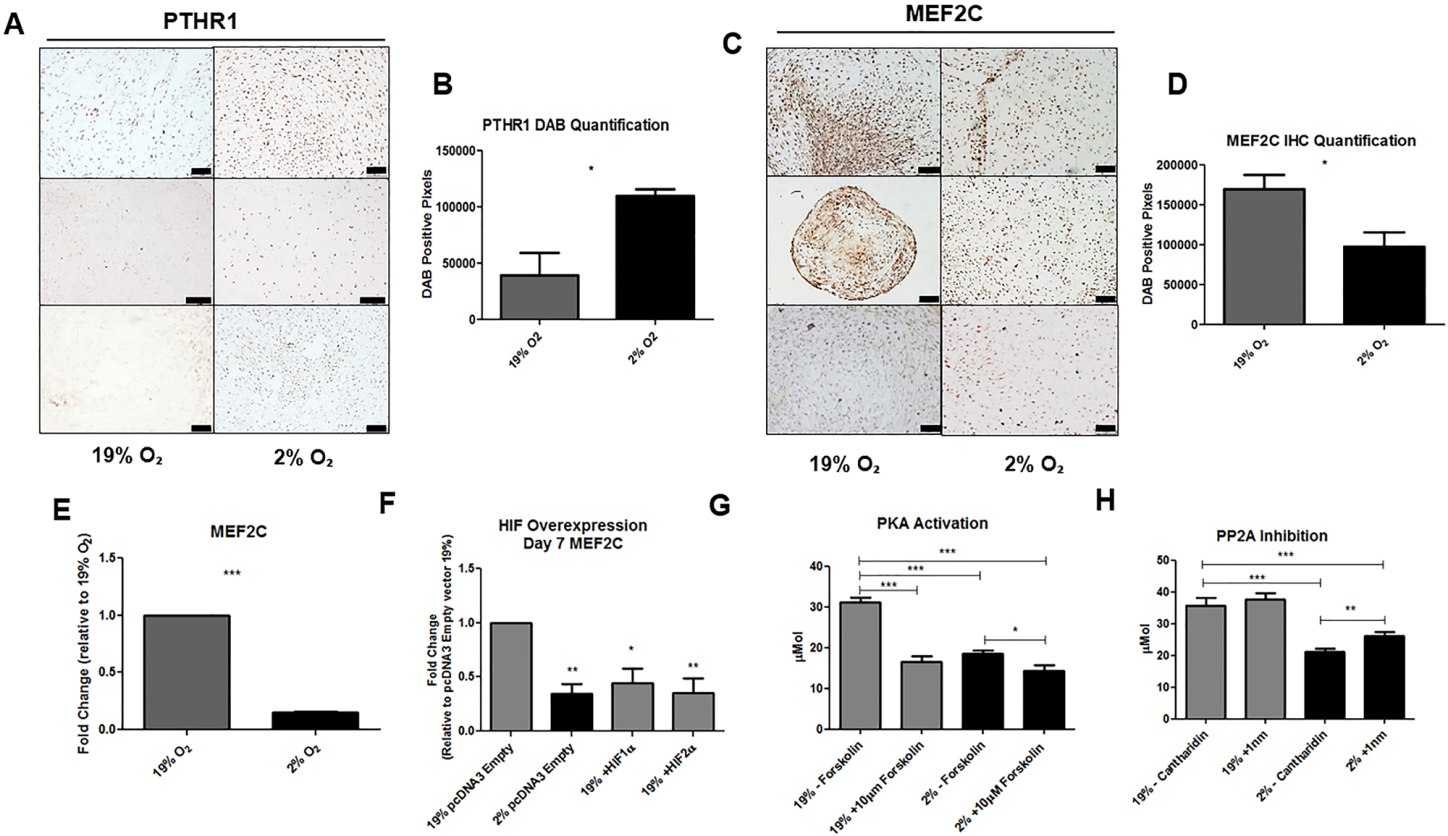
Hypoxia attenuates hypertrophy via a PTHrP mediated reduction of MEF2C. Differentiation of MSC micromass pellets under hypoxic conditions was found to increase PTHR1 protein deposition, as demonstrated by immunohistochemistry performed on day 28 MSC micromass pellets (**A**). Upon quantification of this positive staining, hypoxia was found to significantly increase PTHR1 positive staining under hypoxic conditions (p = 0.0289) (**B**). MEF2C protein deposition was reduced by hypoxia, as demonstrated by immunohistochemistry performed on day 28 MSC micromass pellets (**C**). Upon quantification of the MEF2C positive staining, hypoxia was found to significantly reduce MEF2C positive staining under hypoxic conditions (p = 0.044) (**D**). A significant reduction in MEF2C gene transcript was observed in ATDC5 that were differentiated under hypoxic conditions at day 21 when normalized to normoxic controls (p = < 0.001) (**E**). Following transient overexpression of HIF1α and HIF2α in ATDC5 for 7 days, a significant attenuation in MEF2C expression was observed in ATDC5 differentiated under hypoxia conditions that had been transfected with a pcDNA3 empty vector, compared to nomoxic pcDNA3 empty vector controls. ATDC5 that had been transfected with HIF1α and HIF2α overexpression plasmids under normoxic conditions also demonstrated a significant reduction in MEF2C expression (**F**). The PKA activator Forskolin was added to differentiating ATDC5 from day 7 until the endpoint of day 14. Stimulation of PKA by Forskolin significantly reduced ALP concentration in normoxia when compared to vehicle controls (p = < 0.001), and an additive effect was also observed when hypoxia was combined with Forskolin treatment (p = 0.0160) (**G**). The PP2A inhibitor Cantharidin was administered from day 1 until the endpoint of day 14 to differentiateATDC5. Administration of Cantharidin was shown to increase ALP content in hypoxia when compared to vehicle controls (p = 0.0024). Administration of Cantharidin had no effect on cells differentiated under normoxic conditions (**H**). Scale bars = 100 µm.

We wanted to determine if hypoxia reduced hypertrophy via the PTHrP-PTHR1-MEF2C pathway. We observed an increase in PTHR1 immunohistochemical staining in day 28 MSC pellets in hypoxic conditions, indicating that PTHR1 is upregulated by hypoxia (Fig. 4A). Moreover, upon quantification of DAB staining, hypoxia was found to significantly increase PTHR1 deposition (Fig. 4B). Immunohistochemistry revealed MEF2C protein deposition was reduced in response to hypoxia when compared to normoxic controls in human MSC (Fig. 4C). Additionally, upon quantification of DAB staining, hypoxia was found to significantly decrease MEF2C deposition (Fig. 4D). Similarly in ATDC5, hypoxia was found to attenuate MEF2C gene expression at day 21 of differentiation (Fig. 4E). To assess whether the observed repression of MEF2C by hypoxia was mediated by HIFs, we overexpressed HIF1α and HIF2α in ATDC5 for 7 days and observed a reduction in MEF2C expression under hypoxic conditions, when compared to normoxia (Fig. 4F). Under normoxic conditions, the overexpression of both HIF1α and HIF2α also reduced MEF2C gene expression to comparable levels as hypoxic controls (Fig. 4F). Hence, hypoxia has been observed to increase both PTHrP and PTHR1 while reducing MEF2C expression.

Hypoxia activates Protein Kinase A (PKA), resulting in Protein phosphatase 2A (PP2A) upregulation, histone deacetylase 4 (HDAC4) dephosphorylation and subsequent entry into the nucleus inhibiting MEF2C activity. As a result, hypertrophy markers such as Collagen X are attenuated^30^. To assess if hypertrophy could be attenuated by stimulation of PKA, 10 μM of Forskolin (a potent activator of cAMP, which in turn stimulates PKA^33^) was added to ATDC5 media from day 7 to day 14 of differentiation. The addition of Forskolin reduced the ALP content in the CM of ATDC5 differentiated under normoxic conditions to levels comparable to hypoxia alone. The combination of Forskolin and hypoxia demonstrated an additive effect, with a significant reduction in ALP content when compared to hypoxia alone (Fig. 4G). This data suggests the PTHrP- MEF2C signalling pathway can be augmented by direct stimulation of PKA. Development of drug regimens which target PKA may be used to activate the PTHrP-MEF2C pathway without the use of physiological hypoxia.

Secondly, we wanted to establish if hypertrophy is enhanced by inhibition of PP2A. To do so, we added 1 nM of the PP2A inhibitor Cantharidin^34^ to ATDC5 cells from day 1 until day 14 of differentiation. We observed a significant increase in ALP content in ATDC5 cells differentiated in hypoxia compared to vehicle controls. There was no significant effect observed in normoxia. Overall these data indicates that the hypoxia mediated reduction in ALP is regulated by PP2A.

### FG-4592 treatment attenuates markers of hypertrophy and maintains high levels of GAG synthesis

**Figure 5:**
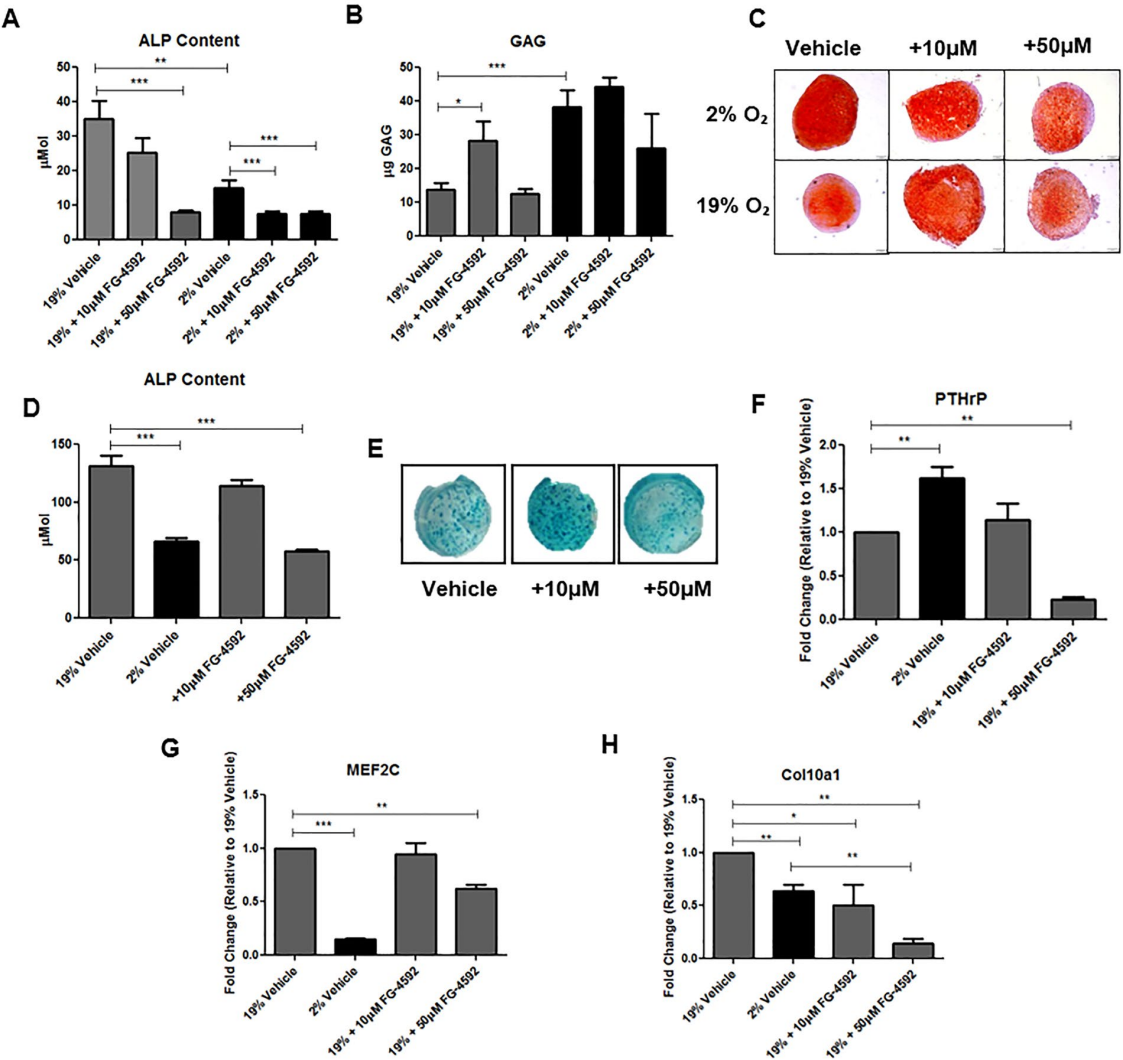
FG-4592 treatment attenuates markers of hypertrophy and maintains high levels of GAG synthesis. Administration of 50 μM FG-4592 reduces ALP content under normoxic conditions at day 28 of differentiation in MSC micromass pellets (p = < 0.001). Under hypoxic conditions, administration of FG-4592 resulted in an additive effect with both 10 μM (p = 0.0025) and 50 μM (p = < 0.001) further reducing ALP content when compared to hypoxic vehicle controls (**A**). Under normoxic conditions, administration of 10 μM FG-4592 significantly improved GAG synthesis at day 28 of differentiation (p = 0.0368). Administration of 50 μM FG-4592 had no significant effect on GAG synthesis in either hypoxic or normoxic conditions (**B**). Safrinin-O staining was performed to visualise GAG deposition in MSC micromass pellets following FG-4592 addition (**C**). ALP content was analysed at day 21 of ATDC5 differentiation following FG-4592 administration. Under normoxic conditions, addition of 50 μM FG-4592 significantly attenuated ALP content when compared to normoxia vehicle controls (p = < 0.001). 10 μM FG-4592 had no significant effect on ALP content (**D**). ATDC5 were stained with Alcian blue to visualize GAG deposition. Positive staining was observed under all conditions, however ATDC5 that had received 50 μM FG-4592 appear to have reduced positive staining when compared to vehicle controls and 10 μM FG-4592 (**E**). QPCR was performed to analyse gene expression following FG-4592 administration. 10 μM FG-4592 had no effect on PTHrP gene expression at 21 days. 50 μM FG-4592 had a detrimental effect on PTHrP gene expression when compared to normoxic vehicle controls (p = < 0.001) (**F**). MEF2C gene expression was significantly reduced by administration of 50 μM FG-4592 when compared to normoxic vehicle controls at day 21 of differentiation (p = < 0.01) (**G**). Col10a1 gene expression was found to be reduced by hypoxia (p = 0.0036), 10 μM FG-4592 (p =  < 0.05), and 50 μM FG-4592 (p = < 0.001), when compared to normoxic vehicle controls (**H**). Scale bars = 200 µm.

The small molecule HIF prolyl hydroxylase inhibitor FG-4592 acts as a hypoxia mimetic by inhibiting the prolyl hydroxylase enzymes that degrade HIF, leading to HIF stabilisation and activation of hypoxia-responsive genes^35^. Treatment of MSC pellets with FG-4592 induced a dose-dependent decrease in ALP content under normoxic conditions. We observed an additive reduction in ALP activity when FG-4592 treatment was combined with hypoxic differentiation of MSC (Fig. 5A). FG-4592 treatment also increased GAG deposition in pellets cultured under normoxic conditions (Fig. 5B), and this improvement in GAG deposition was also observed by Safranin-O staining (Fig. 5C). Similar effects were observed in ATDC5 cells treated with FG-4592. ALP content decreased in a dose-dependent fashion under normoxic conditions (Fig. 5D), and a dose of 50 μM FG-4592 induced a minor decrease in positive Alcian blue staining. However, the 10 μM dose of FG-4592 increased Alcian blue staining under normoxic conditions (Fig. 5E). PTHrP gene expression was significantly inhibited by the high dose (50 μM) of FG-4592, while the lower dose (10 μM) has no significant effect of PTHrP gene expression (Fig. 5F). Conversely, the high dose of FG-4592 significantly attenuated MEF2C expression under normoxic conditions when compared to vehicle controls (Fig. 5G); thus indicating that MEF2C expression is attenuated by FG-4592 treatment. FG-4592 reduced Col10a1 expression at both the high and low doses. 10 μM FG-4592 reduced Col10a1 expression by ~50%, and 50 μM FG-4592 reduced Col10a1 expression by ~85%. The addition of the high dose of FG-4592 also reduced Col10a1 when compared to both normoxic vehicle and hypoxic vehicle controls. This indicates that the administration of FG-4592 causes downregulation of Col10a1, a key marker of hypertrophic differentiation in cartilage (Fig. 5H). This data suggests that 10 μM FG-4592 administration results in increased GAG deposition in MSC pellets under normoxic conditions but the 50 μM dose is required to have a significant effect on hypertrophy.

## Discussion

MSCs have been touted as a possible cellular source for the regeneration of damaged cartilage in recent years, however the proclivity for MSC derived cartilage to undergo hypertrophy *in vivo*^5,8^ remains a concern. This study aims to assess if this concern can be alleviated by using hypoxia to attenuate hypertrophy, potentially improving the fate of an MSC graft *in vivo* following transplantation into a cartilage defect.

Our results demonstrate, for the first time in MSCs, the roles of PTHrP and MEF2C in attenuating hypertrophy in response to hypoxic conditioning. We show that PTHrP can be regulated in response to hypoxia as demonstrated by the increase in PTHrP secretion from MSC pellets and PTHrP gene expression in ATDC5.

It has been reported that the addition of PTHrP to MSC-derived cartilage reduces markers of hypertrophy *in vitro*, however PTHrP also reduces Collagen II expression^6,36^. In our study, we observed that addition of PTHrP can have a negative effect on GAG synthesis, with a dose-dependent reduction in GAG in cartilage differentiated under normoxic conditions. However, by combining hypoxia and the addition of exogenous PTHrP, we observed an additive effect, whereby high levels of GAG synthesis were maintained while reducing ALP activity to basal levels. Notably, when compared to normoxia, 100-fold less PTHrP peptide was required to induce a significant reduction in ALP activity in hypoxia. We postulate that there may be two reasons for this effect: First, we observed that hypoxia induced expression of the PTHrP receptor PTHR1, which may increase the cellular response to low doses of PTHrP. Secondly, we have shown hypoxia increases PTHrP secretion in MSC pellets, thus we expect the increase in endogenous PTHrP to supplement the exogenous PTHrP peptide, leading to a synergistic effect. These results indicate that maintenance of PTHrP and PTHR1 expression in MSC *in vivo* may yield a favourable effect on an MSC graft in a cartilage defect or OA animal model, however further work is required to test this hypothesis *in vivo*.

Downstream of PTHrP, we observed for the first time the hypoxic regulation of MEF2C. MEF2C is a transcription factor that has been shown to be downregulated in response to PTHrP and Forskolin, and can repress Col10a1 gene activation^30^. In addition, knockdown of MEF2C in osteoblasts results in the attenuation of genes associated with hypertrophic and osteoblastic differentiation^32^. Upon activation of the PTHrP receptor PTHR1, PKA activates PP2A, which in turn is responsible for the dephosphoralation of HDAC4. Following the nuclear localization of HDAC4, expression levels of MEF2C are attenuated^30^.

Here we observe that, under hypoxic conditions, MEF2C gene transcript and protein levels are decreased in ATDC5 when compared to normoxic controls. In order to demonstrate that markers of hypertrophy can be reduced by direct stimulation of PKA we added Forskolin to the differentiation systems. Forskolin is a potent activator of cAMP, which in turn stimulates PKA. This results in downregulation of MEF2C expression^30^. Following addition of Forskolin to ATDC5, we noted a significant decrease in ALP activity following 14 days of differentiation in normoxia, indicating that direct stimulation of cAMP/PKA/MEF2C with Forskolin is sufficient to reduce ALP activity. In order to demonstrate that PP2A is necessary for hypoxia-induced attenuation of hypertrophy, we blocked PP2A with Cantharidin, a specific and potent PP2A inhibitor^34^. The addition of Cantharidin to differentiating ATDC5 under hypoxic conditions resulted in a significant upregulation in Alkaline phosphatase content in CM compared to vehicle controls. These results indicate that activation of PP2A is necessary for the attenuation of hypertrophy markers by hypoxia.

Having established the importance of the PTHrP - MEF2C pathway in reducing hypertrophy, our next goal is to develop a treatment regime to inhibit hypertrophy for use in clinical applications. FG-4592 is pharmacological hypoxia-mimetic that is currently undergoing phase III clinical trials to treat anaemia associated with chronic kidney disease (Clinicaltrials.gov ID# NCT01887600). We can report a significant increase in GAG synthesis in pellets treated with 10 μM FG-4592 under normoxic conditions following the addition of FG-4592 to MSC pellets undergoing chondrogenesis. QPCR revealed that FG-4592 treatment can have aberrant effects on gene expression, as expected mRNA transcript levels of MEF2C and Col10a1 were reduced by high doses of FG-4592. However, unexpectedly PTHrP gene transcript levels were also reduced. We postulate that the lack of hypertrophy observed following administration of 50 μM FG-4592 may feedback into an inhibition of PTHrP, although subsequent studies will be required to confirm this. As we observed in our study, 10 μM FG-4592 increases GAG synthesis and 50 μM FG-4592 decreases hypertrophy, therefore a dose escalation study moving from the low to high dose of FG-4592 over time may be beneficial in improving GAG synthesis while subsequently significantly attenuating markers of hypertrophy.

However, it must be noted that while the authors feel that strong evidence has been provided to demonstrate that hypoxia-induced stimulation of the PTHrP – MEF2C pathway inhibits hypertrophy, a limitation of this study is that genetic manipulation studies were only performed in ATDC5 cells and not MSCs. The authors had attempted to use a lentiviral shRNA system to knock-down key genes of interest in MSCs, however we were unable to generate the large numbers of transfected and viable cells required to perform the necessary micro-mass pellet studies. The greater specificity of a CRISPR-Cas9 system^37^ to genetically manipulate cells may be advantageous for future studies which seek to knock-down MEF2C for example. As ATDC5 cells have been shown to demonstrate sequential chondrogenic differentiation, GAG deposition followed by the upregulation of hypertrophy markers^15–17^, we envisage that the results observed in the ATDC5 cell line would readily translate to primary cells in a 3D system however further studies are required to confirm this hypothesis.

In conclusion, this study has demonstrated for the first time that hypoxia stimulates PTHrP and reduces MEF2C to attenuate hypertrophy in mesenchymal stem cell derived cartilage. Further understanding of the pathways described herein may permit the development of a therapeutic MSC product that can produce hyaline cartilage and that is resistant to hypertrophy *in vivo*.

## Methods

### Mesenchymal stem cell isolation and expansion

All procedures were ethically approved by the Clinical Research Ethical Committee at University College Hospital, Galway. Informed constant was obtained from all donors for study participation. All experiments were performed in accordance with relevant guidelines and regulations. Human bone marrow MSCs were isolated from bone marrow harvested from the iliac crest of healthy donors (18–30 years) with approval from the University College Hospital Galway ethics committee (Approval number - CA02/08). Bone marrow derived MSCs were expanded by direct plating technique as previously described^38^. MSC expansion media consisted of alpha MEM (Gibco), 10% Hyclone fetal bovine serum (FBS) (Thermo Scientific), 1% penicillin/streptomycin (Sigma) and 5 ng/ml fibroblast growth factor 2 (FGF2; Pepro-Tech). MSCs were then cultured at 2% oxygen (hypoxia) in a hypoxia incubator (Galaxy RS hypoxia incubator; New Brunswick) or 19% oxygen (normoxia). Chondrogenesis was initiated between passages 2 and 4.

### Micromass pellet chondrogenic differentiation of MSC

3D pellet chondrogenic differentiation^39^ was initiated with 250,000 cells per pellet. MSCs were resuspended in complete chondrogenic media (CCM) - DMEM high glucose (Sigma), 100 nM dexamethasone, 50 μg/ml ascorbic acid 2- phosphate, 40 μg/ml L-proline, 1% ITS supplement (Becton Dickinson), 1 mM sodium pyruvate (Gibco), 1% penicillin/streptomycin (Sigma) with 10 ng/ml transforming growth factor β-3 (TGFβ3) (Pepro-Tech). Pellets from MSCs expanded in hypoxic conditions were differentiated in hypoxia and MSCs expanded at normoxia were differentiated in normoxia. Pellets were differentiated for 28 days, with CCM replaced 3 times/week. Conditioned media (CM) was harvested at days 7, 14, 21 and 28 of differentiation.

To assess the effect of exogenous PTHrP (1–34), increasing concentrations (10 pg/ml, 100 pg/ml, 1 ng/ml, 10 ng/ml, 100 ng/ml) of PTHrP (1–34) peptide (Bachem) were added to CCM 3 times/week from days 14–28.

FG-4592 (Stratech Scientific) was added (10 µM or 50 µM) from day 1 to day 28 of human MSC differentiation or to day 21 for ATDC5. FG-4592 was added every 2 days. DMSO was used as a vehicle control.

### Glycosaminoglycan (GAG) and DNA quantification of micromass pellets

Pellets were digested by Papain (Sigma) overnight at 60 °C. GAG quantification was performed using a 1, 9 dimethylmethylene blue (DMMB) assay as previously described^38^. Quantification of dsDNA in the digested pellets was performed using a Quant-iT Pico Green dsDNA kit (Invitrogen) according to the manufactures protocol. The combination of results from the DMMB and pico-green assays provides a ratio of GAG normalized to dsDNA content.

### Alkaline phosphatase (ALP) assay

CM from day 28 pellets was assayed to quantify ALP content. 10 μl of CM was combined with 190 μl of pNPP substrate (Sigma), incubated for 30 minutes and absorbance analysed at 405 nm and converted to μM using the equation: Absorbance = (extinction coefficient)(concentration)(pathlength).

### PTHrP (1–34) Immunoassay

To quantify secretion of PTHrP (1–34) a PTHrP immunoassay (Bachem) was performed as per the manufacturer’s instructions.

### Safranin-O staining for GAG

Pellets were fixed in 10% Formalin (Sigma) and processed using a Leica ASP300 processor. Paraffin-embedded samples were sectioned at 5 μm and mounted. Samples were deparafinized in Histoclear (National Diagnostics) and rehydrated in decreasing alcohol series. Sections were then incubated in Haematoxylin (Sigma), 0.02% Fast Green (Sigma) and 1% Safranin-O (Sigma), and dehydrated in an increasing alcohol series.

### Immunohistochemistry

Antigen retrieval was performed in 10 mM sodium citrate buffer (pH 6.0). When staining for Collagen II and Collagen X, samples were incubated in 40mU/ml chondroitinase ABC (Sigma). Primary antibodies were diluted in 10% goat serum at concentrations detailed in Table 1. Staining was developed using DAB + Chromogen (Abcam).

**Table 1:** Details of antibodies, concentrations and incubation times used to analyse protein deposition in chondrogenic MSC pellets by immunohistochemistry.

Antibody	Manufacturer	Chondroitinase ABC Treated	Antibody Concentration	Incubation
Collagen II	Abcam #54236	Yes	Pre-diluted	4 °C 18 hrs
RUNX2	Abcam #76956	No	1:100	37 °C 1 hr
MEF2C	Abcam #64644	No	1:200	4 °C 18 hrs
Collagen X	Abcam #49945	Yes	1:1000	4 °C 18 hrs
PTHR1	Abcam# 104832	No	1:200	4 °C 18 hrs

### Quantification of immunohistochemistry

The open-source imaging software Image J with the associated plugin IHC Profiler^40^ was used in order to quantify the extent of positive staining observed in immunohistochemical sections (http://imagej.net/). Briefly, this plugin allows for the colour deconvolution of the image of interest. Once the DAB specific pixels were separated from the rest of the image, the threshold values were manually set to ensure the all DAB staining was accounted for while removing any remaining background/non-specific staining. After the threshold had been set, the “analyse particles” function of Image J was selected. A report was automatically generated by Image J containing the details of the number of DAB positive regions in the image along with the number of pixels contained within each positive region.

### ATDC5 cell culture

ATDC5 cells^14^ were obtained from the European Collection of Cell Cultures (ECACC) and expanded in DMEM/Ham’s F12 + Glutamine (Sigma), 5% FBS, 1% penicillin/streptomycin. Cells were expanded at either hypoxia in a 2% hypoxia incubator or normoxia for at least 1 passage before differentiation was initiated.

### Monolayer chondrogenic differentiation of ATDC5 cells

ATDC5 were seeded at 12,500 cells/cm² and differentiated at both hypoxia and normoxia for 21 days. Differentiation media consisted of DMEM high glucose (Sigma), 100 nM dexamethasone, 50 μg/ml ascorbic acid 2- phosphate, 40 μg/ml L-proline, 1% ITS supplement, 1 mM sodium pyruvate, 1% penicillin/streptomycin with 2% FBS.

To examine the role of the PKA pathway during ATDC5 differentiation, 10 μM of Forskolin was added to ATDC5 media from day 7 to day 14 of differentiation. Also, 1 nM of the PP2A inhibitor Cantharidin was added to ATDC5 media from day 1 until day 14 of differentiation. Both Forskolin and Cantharidin were suspended in DMSO. Equal amounts of DMSO were added as vehicle controls to ATDC5s not receiving the compounds.

### HIF overexpression in ATDC5

HIF1α and HIF2α pcDNA3 triple mutant plasmids (Addgene HIF1α plasmid #44028 and HIF2α plasmid #44027) encode an oxygen-regulation insensitive (normoxia-stable and active) full-length murine HIF1α or HIF2α^41^. ATDC5 were seeded at ~10,000 cells/cm² in expansion media for 24 hours. Prior to transfection, plasmid DNA, FuGENE HD (Promega), and OptiMEM media (Invitrogen) were combined and incubated for 10 minutes before adding to the ATDC5. ATDC5 were incubated for 48 hours or 7 days and cells harvested for subsequent QPCR analysis.

### RNA isolation, cDNA synthesis and quantitative PCR

RNA isolation was performed using Trizol (Sigma) and an RNeasy Mini Kit (Qiagen) according to the manufacturer’s instructions. cDNA was generated using a cDNA synthesis kit (Bioline) according to manufacturer’s protocol. Gene expression was analysed using TaqMan probe sets (Applied Biosystems) details of the TaqMan assays used can be found in Table 2. QPCR was performed on a StepOnePlus instrument (Applied Biosystems) to obtain comparative ΔΔCt values. Data was normalized to the non-hypoxia responsive housekeeping gene RPLP0.

**Table 2:** TaqMan gene expression assay details.

Gene	TaqMan Assay Number
RPLP0	Mm00725448_s1
PTHrP	Mm00436057_m1
MEF2C	Mm01340842_m1
RUNX2	Mm00501584 m1
Col10a1	Mm00487041_m1

### Statistical analysis

All MSC experiments are presented as the mean of a minimum of 3 different human MSC donors ± SEM in order to take into account the biological variation observed across human donors. In addition, a minimum of 3 biological replicates were performed for each individual MSC donor.

All ATDC5 experiments are presented as a minimum of 3 separate and independent experiments. In addition, a minimum of 3 biological replicates per independent experiment were also included and analysed.

To determine statistical significance, a two-tailed Student’s T-TEST was performed when comparing only 2 groups. When comparing more than 2 groups, a one-way ANOVA was performed followed by Tukey’s Multiple Comparison Test to compare differences between groups. Statistically significant changes are marked as *p < 0.05; **p < 0.01; ***p < 0.0001. Statistical analysis was performed using GraphPad Prism 6 (GraphPad Software).

## Data Availability

The datasets generated during and/or analysed during the current study are available from the corresponding author on reasonable request.
